# Identification and Characterization of Olfactory Genes in the Cochineal Scale Insect, *Porphyrophora sophorae* (Hemiptera: Margarodidae)

**DOI:** 10.3390/biology14101442

**Published:** 2025-10-18

**Authors:** Yan Wang, Xiao-Li Liu, Youssef Dewer, Cai-Ge Jiang, Shuang Song, Hong-Hao Chen

**Affiliations:** 1 Institute of Plant Protection, Ningxia Academy of Agriculture and Forestry Sciences, Yinchuan 750002, China; wangyan105422@163.com (Y.W.); xiaoli_8302@163.com (X.-L.L.); jiangcaige168@126.com (C.-G.J.); wagg1987@163.com (S.S.); 2 Phytotoxicity Research Department, Central Agricultural Pesticide Laboratory, Agricultural Research Center, 7 Nadi El-Seid Street, Giza 12618, Egypt; dewer72@yahoo.com

**Keywords:** *Porphyrophora sophorae*, antenna transcriptome, odorant binding proteins, olfactory receptors, tissue expression

## Abstract

**Simple Summary:**

To investigate the olfactory recognition mechanisms of *Porphyrophora sophorae*, this study sequenced the adult antennae transcriptome using the PacBio RS II platform. We identified 11 OBP and 11 OR genes, with seven PsopOBPs and one PsopOrco showing significant overexpression in male antennae. These findings reveal the molecular basis of volatile compound recognition from licorice plants in *P. sophorae*, providing potential targets for eco-friendly pest management strategies.

**Abstract:**

Chemosensory systems are essential in insect behavior, with several key genes associated with these systems emerging as potential targets for pest control. *Porphyrophora sophorae* (Archangelskaya, 1935), a destructive pest of Chinese licorice (*Glycyrrhiza uralensis*, Fabaceae), poses a significant threat to the healthy cultivation of licorice. However, the molecular mechanisms underlying its host detection and olfactory recognition remain poorly understood. In this study, we present the first identification of odorant-binding proteins (OBPs) and olfactory receptors (ORs) from the transcriptome of *P. sophorae*. The identified OBPs contain six conserved cysteine residues, while predictive analysis suggests that *PsopOrco* may contain six transmembrane domains. Phylogenetic analysis demonstrated that the majority of these olfactory proteins are closely related to OBPs and ORs found in other scale insects. Using RT-qPCR, we assessed the anatomical structures expression of these genes and found that *PsopOBP3*, *PsopOBP6*, and *PsopOrco* were predominantly expressed in the antennae. Additionally, expression levels of OBPs and ORs varied across different tissues, suggesting anatomical structure regulation. These findings expand the gene repertoire of *P. sophorae* and provide valuable resources for further functional analysis of these key olfactory genes.

## 1. Introduction

Olfaction plays a crucial role in the life cycle of insects, including host location, feeding, communication, and reproduction, which ultimately contribute to population growth [1,2]. The olfactory detection and signal transduction in insects involve a complex array of proteins. These proteins mediate the detection, binding, transport, recognition, activation, transduction, and degradation of environmental chemical signals. Ultimately, these processes enable the transmission of olfactory signals to the insect’s nervous system [3,4]. Investigating the olfactory genes provides insights into the molecular mechanisms through which chemical signals are converted into electrical signals within the olfactory nerves. Previous research on olfactory genes in pests also contributes to understanding the co-evolutionary dynamics between pests and their host organisms. Moreover, targeting olfactory systems in pest management, including the identification of sex pheromones using reverse chemical ecology and structural biology, offers potential strategies for environmentally friendly pest control [5,6,7].

Insects primarily rely on their antennae, which are covered with diverse sensory structures, as sensory organs for detecting external olfactory cues. These sensilla contain pores that allow odorants to enter, forming the first barrier of recognition for chemical signals [8]. Insect olfactory processing requires multiple key protein families. These include odorant-binding proteins (OBPs), chemosensory proteins (CSPs), olfactory receptors (ORs), ionotropic receptors (IRs), odorant-degrading enzymes (ODEs), and sensory neuron membrane proteins (SNMPs) [9,10,11]. OBPs are essential in the peripheral olfactory system, while ORs are central to the chemosensory signaling pathway. The recognition of odor molecules by ORs is a pivotal step in this process.

Insect olfactory binding proteins (OBPs) possess characteristics such as small molecular weight and ease of recombinant expression, making them ideal subjects for identifying key chemical features through ligand screening. These properties give OBPs significant advantages in developing behavioral regulators for pests, positioning them as preferred targets for green pest control strategies [12]. Meanwhile, insect olfactory receptors (ORs) demonstrate more precise ligand specificity, particularly exhibiting highly selective responses to pheromones. The direct correlation between their activation or inhibition and specific behaviors makes ORs ideal targets for intervening in pest behavior. Given the critical roles and targeting potential of OBPs and ORs in the pest olfactory system, this study will focus specifically on in-depth research of these two protein types [12].

OBPs and ORs are the most studied olfactory-related proteins. Odor molecules entering the sensilla interact with OBPs, which initiate the first stage of olfactory signal detection [13,14]. OBPs are not only highly expressed in insect antennae but are also found in other tissues such as labial palps, proboscis, stylets, abdomen, legs, wings, ovipositor, and gonads, suggesting broader physiological functions beyond olfaction [15]. When hydrophobic odorant molecules diffuse into the antennal sensilla, they are specifically captured and bound by OBPs in the sensillar lymph. The OBPs then transport the odorants to the vicinity of either olfactory receptors (ORs) or ionotropic receptors (IRs) located on the dendritic membranes of olfactory receptor neurons (ORNs). OBPs identified across various insect species exhibit considerable divergence, with significant variation between different insect taxa [16].

At the receptor level, insect ORs exhibit significant evolutionary diversity, though their degree of conservation varies across taxa: homologous ORs among closely related species or within the same species can maintain 80–90% sequence similarity, while certain rapidly evolving OR subfamilies (such as *Drosophila* pheromone receptors) may show even lower identity [17]. ORs have been identified in various species, including *Drosophila melanogaster* [17], *Bombyx mori* [18], *Apis mellifera* [19], *Anopheles gambiae* [20], and *Harmonia axyridis* [21]. Notably, all typical ORs depend on a highly conserved co-receptor, Orco (Odorant receptor co-receptor), to form heteromeric complexes. In this complex, Orco is responsible for the functional assembly of the ion channel, while the variable receptor subunit determines ligand specificity. This unique mechanism makes Orco an ideal target for studying insect olfactory regulation. Advancements in high-throughput transcriptome sequencing have greatly facilitated the study of olfactory-related genes. Following the release of the *Melitaea protomedia* Méniétriés transcriptome [22], a large number of insect antennal transcriptomes have been sequenced, leading to the identification of olfactory-related genes. In Hemipteran piercing-sucking insects, olfactory-related genes have been identified in species such as *Empoasca onukii* [23], *Apolygus lucorum* [24,25], *Cacopsylla chinensis* [26], *Myzus persicae* [27], and *Riptortus pedestris* [28]. In stark contrast, research on scale insects—a group encompassing numerous globally significant pests—remains critically understudied, with olfactory-related proteins reported in only two species to date [29,30,31]. This significant knowledge gap severely limits our understanding of the molecular mechanisms underlying chemical communication in scale insects and hinders the development of targeted, sustainable pest management strategies. Elucidating the olfactory mechanisms of scale insects is therefore imperative, not only to advance fundamental knowledge of insect olfaction but also to identify species-specific molecular targets for novel eco-friendly control approaches, such as behavior-disrupting techniques based on olfactory interference.

Building upon the broader context of insect olfactory systems and the identified research gaps in scale insects, we turn to *Porphyrophora sophorae* as a critical case study. *This* devastating pest of Chinese licorice (*Glycyrrhiza uralensis*, Fabaceae) represents a major threat to the healthy cultivation of this important medicinal plant. The ovisacs of *P. sophorae* parasitize the rhizomes of licorice plants, using their stylets to pierce the phloem and absorb nutrients, leading to root rot and plant withering due to fungal invasion [32]. *P. sophorae* significantly impacts the quality and yield of licorice stems, earning it the nickname “the cancer of licorice” [33]. Control strategies for *P. sophorae* have long relied heavily on chemical pesticides. However, these have not only failed to provide effective long-term suppression of pest populations but have also led to undesirable residues in medicinal materials and the accumulation of toxic heavy metals in the cultivation environment. These outcomes collectively undermine the competitiveness and sustainability of the licorice industry. Consequently, there is an urgent need for environmentally friendly pest control measures. In recent years, research on *P. sophorae* has focused on biological studies [34,35], chemical control [36,37], antennal sensilla morphology [38,39], and host-location behavior [40], but no research has yet been conducted on its chemical ecology.

In this study, we sequenced and analyzed the adult antenna transcriptome of *P. sophorae* for the first time using PacBio sequencing. We identified OBPs and ORs in *P. sophorae*, performed sequence alignment and phylogenetic analysis, and assessed their expression in various tissues using RT-qPCR. While this work primarily focuses on gene identification and expression patterns, the distinct overexpression of specific OBPs and ORs in male antennae provides crucial clues for their potential functional roles in host recognition and mate-seeking behaviors. These findings not only establish a molecular foundation for understanding the olfactory mechanisms in *P. sophorae* but also pinpoint candidate genes for future functional investigations, such as ligand binding assays and RNAi-mediated behavioral studies, which could ultimately contribute to developing behavior-based control strategies against this destructive pest.

## 2. Materials and Methods

### 2.1. Insects and Sample Collection

Adults of *P. sophorae* used in this study were collected from a licorice field located in Tianjizhang village, Huamachi Town, Yanchi County, NingXia Hui Autonomous Region, China (107°18′36″ N, 37°48′00″ E, elev.1600 m), in August 2021. To construct a comprehensive transcriptome, antennae from healthy adult *P. sophorae* were dissected and processed as follows: For PacBio third-generation sequencing, one pooled sample was prepared for each sex, with each sample containing antennae from 100 individuals. For Illumina second-generation sequencing (used for subsequent expression analysis and error correction of PacBio reads), three independent biological replicates were prepared for each sex, with each replicate also consisting of antennae from 100 individuals.

For tissue-specific expression analysis, the following anatomical structures were collected: antennae, head (excluding antennae), thorax, abdomen, and legs. For each tissue type, three biological replicates were obtained. Each biological replicate was pooled from the following numbers of adult insects: (i) antennae: 100 individuals, (ii) heads (without antennae): 80 individuals, (iii) thoraxes: 50 individuals, (iv) abdomens: 50 individuals, and (v) legs: 120 individuals. The dissected samples were immediately frozen in liquid nitrogen and stored at −80 °C for further analysis.

### 2.2. Antennal cDNA Library Construction, Sequencing and Analysis

We employed a hybrid sequencing approach to maximize the advantages of both platforms. PacBio SMRT sequencing was utilized to obtain full-length transcript isoforms without assembly, which is particularly valuable for accurately characterizing gene families like OBPs and ORs that often contain multiple isoforms and close paralogs. Meanwhile, Illumina short-read sequencing provided high-depth coverage for two critical applications: (1) error correction of the PacBio long reads to enhance base-level accuracy, and (2) quantitative expression analysis across different tissues and sexes. This combined approach allowed us to simultaneously achieve comprehensive gene identification and reliable expression profiling.

Total RNA was extracted using the TRIzol (Thermo Fisher Scientific, Waltham, MA, USA) reagent following the manufacturer’s instructions. RNA purity and concentration were assessed using a Nanodrop spectrophotometer (Nanodrop2000, Thermo Fisher Scientific, Waltham, MA, USA), while RNA integrity was evaluated with an Agilent 2100 Bioanalyzer (LabChip GX, PerkinElmer, Waltham, MA, USA). Qualified RNA samples had an OD (260/280) ratio of 1.8–2.2 and an OD (260/230) ratio of <2.0.

Full-length Transcriptome Sequencing (third-generation sequencing). After passing quality control, the cDNA library was constructed as follows: (1) Full-length cDNA was synthesized from mRNA using the SMARTer™ PCR cDNA Synthesis Kit (Clontech, Palo Alto, CA, USA). (2) PCR amplification was performed to amplify the full-length cDNAs. (3) End-terminal repair was conducted on the cDNAs. (4) SMRTbell adapters (SMRTbell Template Prep Kit)(PACBIO, Silicon Valley, CA, USA) were ligated to the cDNAs. (5) Exonuclease digestion was performed to generate sequencing libraries. After library construction, the concentration was measured using a Qubit 2.0 fluorometer (Thermo Fisher Scientific, Waltham, MA, USA), and library size was analyzed with an Agilent 2100 Bioanalyzer. Once the library quality was confirmed, full-length transcriptome sequencing was carried out using the PacBio RS II system, with sequencing services provided by Biomarker Technologies Co. Ltd. (Beijing, China).

NGS Transcriptome Sequencing (second-generation sequencing). After passing quality control, the cDNA library was constructed as follows: (1) mRNA was isolated by Oligo(dT)-attached magnetic beads. (2) mRNA was then randomly fragmented in fragmentation buffer. (3) First-strand cDNA was synthesized with fragmented mRNA as template and random hexamers as primers, followed by second-strand synthesis with addition of PCR buffer, dNTPs, RNase H and DNA polymerase I. Purification of cDNA was processed with AMPure XP beads. (4) Double-strand cDNA was subjected to end repair. Adenosine was added to the end and ligated to adapters. AMPure XP beads were applied here to select fragments within certain size range. (5) cDNA library was constructed by performing 12 cycles of PCR amplification on the cDNA fragments generated in Step 4. The qualified libraries were pooled based on targeted data production and sequenced on Illumina NovaSeq 6000 sequencing platform (Biomarker Technologies Co. Ltd.). A paired-end sequencing strategy was employed, with standard libraries constructed using 350 bp insert fragments (range: 300–400 bp) and sequenced with 2 × 150 bp (PE150) reads. This sequencing parameter design ensures sufficient read length to cover full-length transcripts and meets the data quality requirements for downstream quantitative analysis using RSEM (v1.3.3) and other software.

For the PacBio third-generation sequencing data, we performed the following bioinformatics analyses. Polymerase reads were initially screened, retaining sub-reads that met quality thresholds (>50 bp in length with an accuracy rate > 0.80). These sequences were then processed into circular consensus reads (CCS) based on adapter counts. CCS were categorized into three classes: (1) full-length non-chimeric sequences, (2) full-length chimeric sequences, and (3) non-full-length sequences, determined by the presence of 5′ primers, 3′ primers, and polyA tail signals. For sequence refinement, the SMRT Analysis software (v10.1) suite was employed, specifically using the Iterative Clustering for Error Correction (ICE) algorithm to cluster CCS with high sequence similarity and generate consensus sequences. Subsequent polishing of these consensus sequences incorporated non-full-length reads to enhance sequence quality. The processed transcripts underwent further refinement through CD-HIT 4.6.1 (-c 0.99 -M 0 (cd-hit-est)) clustering (identity threshold > 0.99) to eliminate redundancy, yielding a comprehensive set of full-length PacBio transcriptome sequences. Finally, transcriptome completeness was rigorously assessed using BUSCO [41] (Benchmarking Universal Single-Copy Orthologs) v3.0.2 (-m tran -c 4 -f) against the Insecta_odb10 dataset from OrthoDB. This gene set was selected based on the taxonomic position of our study species within the class Insecta. We performed functional annotation analysis through the following pipeline: First, we aligned the non-redundant transcript sequences against NR, SwissProt, GO, COG, KOG, Pfam and KEGG databases using BLAST software (v 2.2.26). Specifically: (1) Diamond BLASTx (v2.0.15) against NR/Swiss-Prot with E-value cutoff 1 × 10^−5^; (2) HMMSCAN (v3.3.2) for Pfam domain identification; (3) KEGG/COG/KOG assignments via eggNOG-mapper (v2.1.6) using default parameters. GO terms were derived from InterProScan (v 5.34-73.0) analyses.

### 2.3. Gene Expression Analysis

The paired-end reads (PE150) obtained from Illumina sequencing were aligned to the PacBio-assembled transcriptome reference sequences for quantification using RSEM v1.2.19 (-a -m 200) [42].

FPKM (Fragments Per Kilobase of transcript per Million fragments mapped) was used as an indicator to measure transcript or gene expression levels. The FPKM calculation formula is as follows:

FPKM = cDNA Fragments/(Mapped Fragments (Millions) × Transcript Length (kb))

In the formula, cDNA Fragments stands for the number of reads (PE reads) mapped to the specific transcript; Mapped Fragments (Millions) stands for the number of total mapped reads (in 10^6^). Transcript Length (kb): the length of transcripts (in 10^3^ bp).

### 2.4. Identification of Olfactory Genes and Bioinformatic Analysis

Putative olfactory gene transcripts were identified by searching for keywords related to odorant-binding proteins (OBPs) and odorant receptors (ORs) within the annotated unigene dataset. To confirm the identity of these genes, the Blastx and Blastn tools available on NCBI (https://blast.ncbi.nlm.nih.gov/) (accessed on 19 October 2023) were used for further verification. The longest open reading frame (ORF) for each unigene was determined using the ORF Finder tool (http://www.ncbi.nlm.nih.gov/gorf/gorf.html) (accessed on 20 October 2023). Signal peptides for OBP genes were predicted using the SignalP 4.1 server (http://www.cbs.dtu.dk/services/SignalP/) (accessed on 22 October 2023), while transmembrane domains for OR genes were predicted with the TMHMM Server Version 2.0 (http://www.cbs.dtu.dk/services/TMHMM) (accessed on 22 October 2023) and TopCons software (https://topcons.cbr.su.se/) (accessed on 11 April 2025). Sequence alignments of OBPs were visualized using DNAMAN 6.0 (default settings), while Orco sequences were aligned and displayed using PRALINE (PSI-BLAST, 3 iterations, E < 1 × 10^−3^) (http://www.ibi.vu.nl/programs/pralinewww) (accessed on 24 October 2023). Amino acid sequence alignments of mature OBPs and ORs from *P. sophorae* and other Hemipteran species were performed using MAFFT v6.864 (G-INS-I, default settings) (http://mafft.cbrc.jp/alignment/server/clustering.html) (accessed on 25 October 2023). Phylogenetic trees were constructed using the JTT+CAT model with 1000 bootstrap replicates in FASTTREE. The resulting trees were further edited and visualized using iTOL v3 (https://itol.embl.de) (accessed on 26 October 2023).

### 2.5. RT-qPCR Analysis

RT-qPCR was employed to quantify the expression levels of OBPs and ORs in various body tissues (antennae, head (excluding antennae), thorax, abdomen and legs) of adult *P. sophorae*. Total RNA was extracted using the TRIzol reagent, with RNA integrity verified by agarose gel electrophoresis and Nanodrop quantification (A260/A280 ratios between 1.8–2.1). For cDNA synthesis, 1 μg of total RNA from each sample was reverse transcribed using the PrimeScript™ RT Reagent Kit (TaKaRa, Dalian, China) in a 20 μL reaction system, following the manufacturer’s protocol. gDNA Eraser (Perfect Real Time) was used to eliminate genomic DNA contamination. All samples were processed with the same amount of starting RNA (1 μg) to ensure comparability. The RT-qPCR reactions were performed using 2 μL of undiluted cDNA. All qPCR reactions were performed using equal volumes of reverse transcription products to preserve synthesis efficiency variations. Tubulin was used as the internal reference gene for normalization. Gene-specific primers for amplification are listed in Table S1. The RT-qPCR cycling conditions consisted of an initial denaturation at 95 °C for 10 min, followed by 40 amplification cycles of 95 °C for 15 s (denaturation), and 60 °C for 1 min (annealing and extension). The melt curve analysis was performed with an initial step at 95 °C for 5 s, followed by 1 min at 65 °C, a gradual temperature increase to 95 °C (at 0.11 °C/s), and a final step at 50 °C for 30 s. All qPCR experiments strictly followed MIQE guidelines to establish a quality control system: reference gene stability required a coefficient of variation (CV) < 5% across all samples (calculated by the geNorm algorithm); amplification efficiency validation confirmed primer efficiencies between 90–110% using standard curves with regression coefficients (R^2^) ≥ 0.98; technical reproducibility was ensured by triplicate measurements with Ct value standard deviations (SD) < 0.5 cycles; and specificity was verified through single-peak melting curves.

### 2.6. Data Analysis

Three biological replicates were analyzed to assess the relative expression levels of *PsopOBP* and *PsopOR* genes across various anatomical structures using the 2^−(∆∆CT)^ method [43,44]. Differences in gene expression between male and female tissues were evaluated using a one-way nested analysis of variance (ANOVA), followed by Tukey’s Honestly Significant Difference (HSD) test for post hoc comparison. Statistical analyses and graphical representations were performed using GraphPad Prism 8 (GraphPad Software, San Diego, CA, USA), and all data processing was carried out using SPSS (Version 17.0, SPSS Inc., Chicago, IL, USA).

## 3. Results

### 3.1. Overview of the Transcriptome of P. sophorae

To construct the transcriptome of *P. sophorae*, RNA was extracted from adult individuals, and a cDNA library was generated. The library was sequenced using PacBio’s Single-Molecule Real-Time (SMRT) technology on the PacBio RS high-throughput sequencing platform. Circular consensus sequences (CCS) were derived from the original sequences, applying the criteria of full passes ≥ 1 and sequence accuracy > 0.90. This process yielded 412,849 CCS reads (Table S2). The average read length was 910 bp, with a total base count of 375,820,775 and an average of 45 full passes per read. From this, 372,326 full-length non-chimeric (FLNC) reads were obtained. The FLNC sequences were then clustered, resulting in 51,769 consensus isoforms with a mean length of 789 bp (Figure S1). After polishing, 51,764 high-quality consensus isoforms were identified, alongside 5 low-quality consensus isoforms. These low-quality isoforms were corrected using Illumina RNA-seq data and merged with the high-quality FL consensus isoforms. Redundant isoforms were removed, retaining only those with greater than 99% identity, resulting in a final set of 33,579 high-quality, non-redundant FL transcripts. The completeness of the non-redundant FL transcripts was assessed using BUSCO analysis. Out of the 1658 BUSCO groups, 898 were complete (54.2%), including 631 single-copies (S) and 267 duplicated (D) BUSCOs. Additionally, 215 fragmented BUSCOs (13%) and 545 missing BUSCOs (32.8%) were identified (Figure S2). It should be noted that the BUSCO completeness value (54.2%) of our transcriptome, while lower than that of typical model organisms, is consistent with expectations for a non-model species like *P. sophorae* that lacks a reference genome. This is primarily because de novo assembly without a reference genome has inherent limitations in capturing low-abundance transcripts. We acknowledge this partial completeness, which is typical for such datasets; nevertheless, the high-quality sequences obtained were robust for comprehensively identifying the targeted chemosensory gene families (e.g., OBPs and ORs), confirming the utility of our dataset for this study.

### 3.2. Functional Annotation of Non-Redundant High-Quality Transcripts

The non-redundant high-quality transcript sequences were subjected to BLAST alignment against multiple databases, including Nr, SwissProt, GO, COG, KOG, Pfam, and KEGG. This analysis resulted in functional annotations for 16,568 transcripts (Table S3). Compared to other species, a considerable proportion of the annotated transcripts showed relatively high similarity to proteins from *Bemisia tabaci* (Hemiptera: Aleyrodidae), suggesting a close evolutionary relationship between *B. tabaci* and *P. sophorae*. Transcripts from other species demonstrated less than 9% similarity, reinforcing this connection (Figure S3). Gene Ontology (GO) enrichment analysis was performed using tBLASTx with an E-value threshold of <1.0 × 10^−5^, leading to the annotation of 7937 transcripts across various functional categories (Figure S4). Furthermore, 5114 transcripts were assigned to 25 functional categories based on Clusters of Orthologous Groups (COG) analysis (Figure S5). The eggNOG database was employed to provide more detailed functional descriptions of orthologous groups, classifying 15,106 isoforms into 25 subgroups (Figure S6). Additional annotation using the KOG database categorized 11,720 isoforms into broader functional categories (Figure S7). Finally, pathway predictions were made using the KEGG database, identifying 278 pathways containing a total of 9454 transcripts.

### 3.3. Identification of Candidate Olfactory Genes

**Figure 1:**
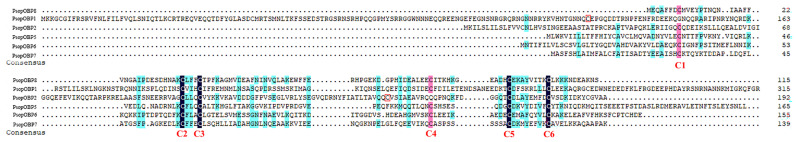
Sequence alignment of odorant-binding proteins (OBPs) from *Porphyrophora sophorae*. Conserved motifs have been highlighted in the figure: Classic OBPs. Red boxes highlight cysteine residues C1 and C4 in *PsopOBP1* and *PsopOBP2*, respectively.

The tBLASTn analysis identified 11 unigenes encoding putative OBP genes in *P. sophorae* (Table 1). Among these, six PsopOBPs exhibited intact open reading frames (ORFs) ranging from 116 to 317 amino acids. Among the 11 OBP genes, three (OBP2, OBP3, and OBP6) were found to contain signal peptides. Sequence alignment revealed that these 11 OBP genes share homology with OBPs from five other insect species: four unigenes showed homology to *Drosicha corpulenta* OBPs, four to *Phenacoccus solenopsis* OBPs, and each of the remaining unigenes shared homology with one OBP from each of the other three species analyzed (Table 1). Based on the conserved ‘classic’ OBP cysteine motif (C1-X20-66-C2-X3-C3-X21-43-C4-X8-14-C5-X8-C6), we classified six of the PsopOBPs (*PsopOBP1–2*, *PsopOBP5–8*) as ‘classic’ OBPs (Figure 1). Notably, among the identified OBPs, only *PsopOBP2* and *PsopOBP6* were found to contain both complete open reading frames and predicted signal peptides, suggesting they represent fully functional secreted proteins.

From the transcriptome of *P. sophorae* antennae, 11 odorant receptor (OR) transcripts were identified (Table 2). Two unigenes (Orco and OR1) exhibited ORF structural features suggestive of completeness, while the remaining shorter sequences (64–108 aa) may represent partial fragments. While the inclusion of these partial sequences may impact the resolution of phylogenetic analysis, they have been retained as they contain conserved key functional domains such as transmembrane regions. It should be emphasized that phylogenetic relationships and structural inferences derived from these incomplete sequences require cautious interpretation. The identified 11 OR unigenes showed homology with those from five other insect species, including six unigenes homologous to *D. corpulenta* ORs, two unigenes homologous to *Ph. Solenopsis* ORs, and one unigene homologous to ORs from each of the remaining species (Table 2). The *PsopOrco* gene exhibited classic odorant receptor characteristics, with an ORF encoding 428 amino acids and showing high conservation compared to homologs from other Hemipteran species (Supplementary Figure S8). The amino acid similarity of *PsopOrco* with Orco orthologs from *Ph. solenopsis*, *Tropidothorax elegans*, *Corythucha ciliata*, *Plautia stali*, *Halyomorpha halys*, and *Melanaphis sacchari* was 96.14%, 63.42%, 61.86%, 62.66%, 62.24%, and 60.74%, respectively (Figure S8).

### 3.4. Phylogenetic Analysis of P. sophorae OBP and OR Genes

**Figure 2:**
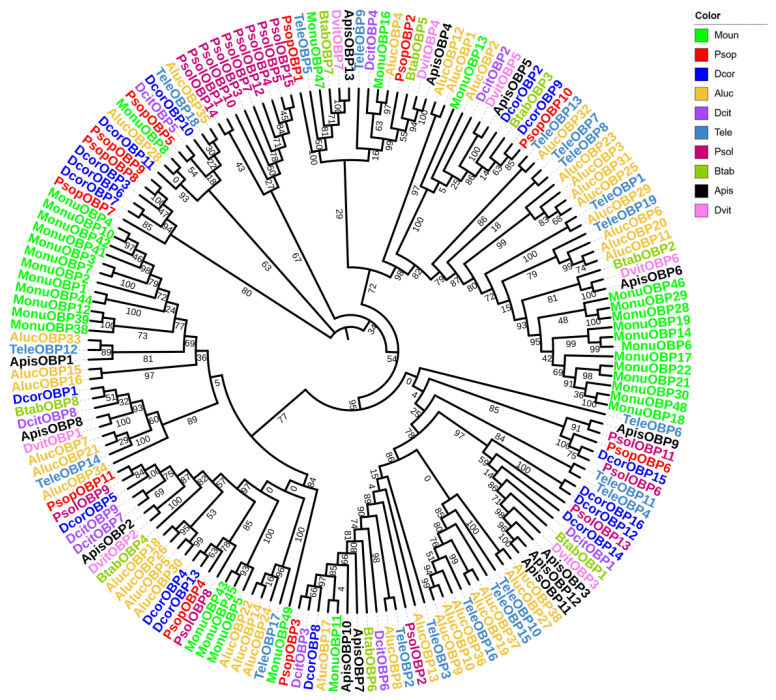
Phylogenetic analysis of OBPs in *Porphyrophora sophorae* (PsopOBPs) in relation to orthologs from nine other Hemipteran species (169 total OBPs). The species included are: Dcor, *Drosicha corpulenta* (*n* = 16), Psol, *Phenacoccus solenopsis* (*n* = 15), Dcit, *Diaphorina citri* (*n* = 9), Btab, *Bemisia tabaci* (*n* = 8); Apis, *Acyrthosiphon pisum* (*n* = 13), Dvit, *Daktulosphaira vitifoliae* (*n* = 7), Aluc, *Apolygus lucorum* (*n* = 38), Tele, *Tropidothorax elegans* (*n* = 19), and Monu, *Matsumurasca onukii* (*n* = 33). The bootstrap values have been represented in the figure.

For the OBP phylogenetic analysis, we selected 169 OBP genes from 10 Hemiptera species, including *P. sophorae*, *D. corpulenta*, *Ph. solenopsis*, *Diaphorina citri*, *B. tabaci*, *Acythosiphon pisum*, *Daktulosphaira vitifoliae*, *Apolygus lucorum*, *T. elegans*, and *Matsumurasca onukii* (Figure 2). The phylogenetic tree showed strong branch support (bootstrap values ≥ 85%), with *PsopOBP7*, *PsopOBP8* and *PsopOBP9* forming a distinct clade, while other OBP genes (*PsopOBP1–6*, *PsopOBP10–11*) showed relatively dispersed phylogenetic relationships. Most of the PsopOBPs were found to cluster with OBPs from the Coccoidea (*D. corpulenta*, *Ph. solenopsis*), suggesting a consistent homologous and inter-species phylogenetic relationship for OBPs across species.

**Figure 3:**
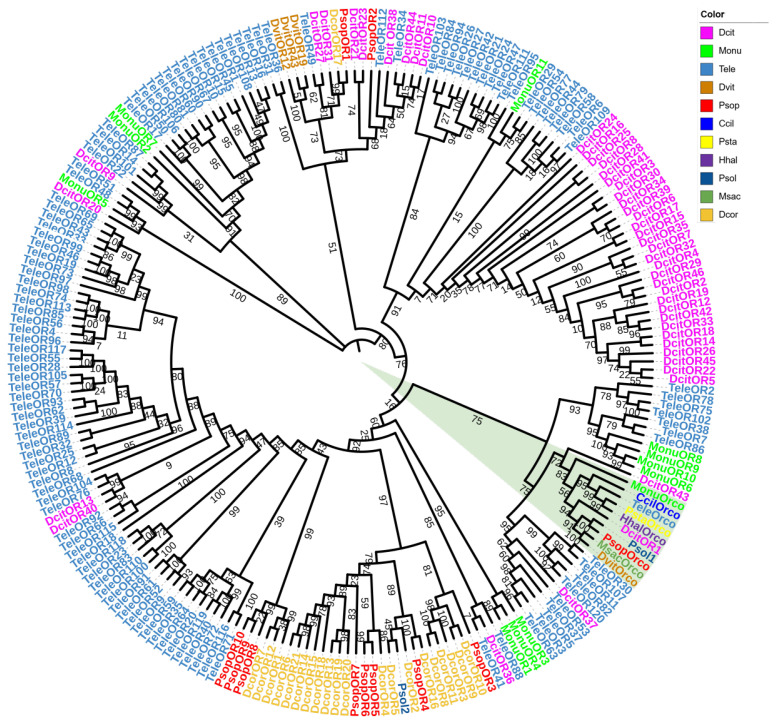
Phylogenetic tree of odorant receptor genes from *P. sophorae* and other Hemipteran species. The species included are: Tele, *Tropidothorax elegans* (*n* = 121), Dcit, *Diaphorina citri* (*n* = 46), Monu, *Matsumurasca onukii* (*n* = 12), Dvit, *Daktulosphaira vitifoliae* (*n* = 4), Dcor, *Drosicha corpulenta* (*n* = 21), Psol, *Phenacoccus solenopsis* (*n* = 2), Ccil, *Corythucha ciliata* (*n* = 1), Psta, *Plautia stali* (*n* = 1), Hhal, *Halyomorpha halys* (*n* = 1), and Msac, *Melanaphis sacchari* (*n* = 1). The bootstrap values have been represented in the figure. Green triangles represent all Orco genes from all species clustered together.

For the OR phylogenetic analysis, 11 *P. sophorae* OR genes and 210 ORs from 10 Hemiptera species (*T. elegans*, *Di. citri*, *M. onukii*, *Da vitifoliae*, *D. corpulenta*, *Ph. solenopsis*, *C. ciliata*, *Pl. stali*, *H. halys*, and *Me. sacchari*) were used to construct the phylogenetic tree (Figure 3). The phylogenetic analysis demonstrated strong overall support, reinforcing the reliability of the tree. Orco genes from all species clustered together (Figure 3, green triangle), while *PsopOR1* and *PsopOR2*, as well as *PsopOR8* and *PsopOR9*, grouped within the same branches. Most of the PsopORs clustered with odorant receptors from other Hemiptera species, indicating consistent homologous and inter-species phylogenetic relationships for ORs across the group.

### 3.5. Anatomical Structures Expression of P. sophorae OBP and OR Genes

**Figure 4:**
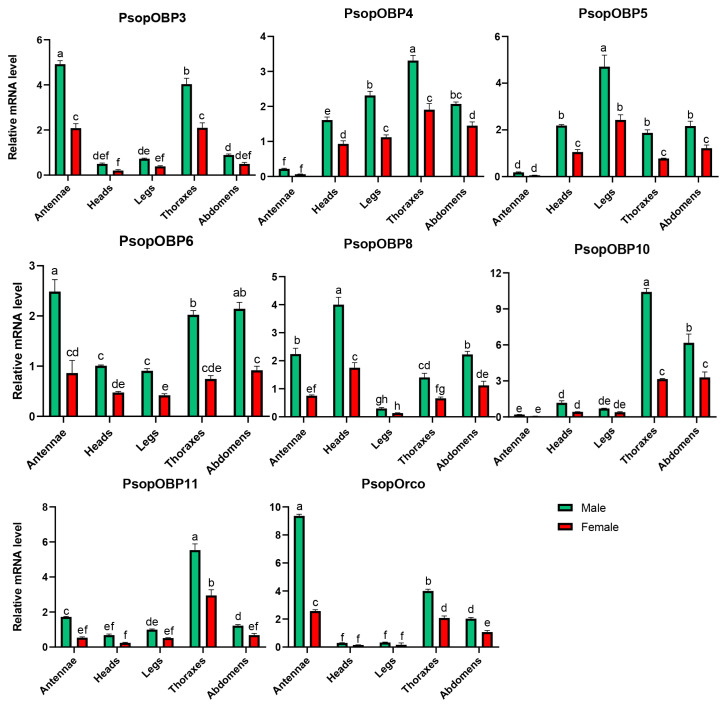
Relative expression of *PsopOBP* and *PsopOR* genes in male and female adult tissues of *P. sophorae*, as determined by RT-qPCR. The relative expression level is indicated as mean ± SE (*n* = 3). Different lowercase letters (a, b, c, etc.) above the bars indicate statistically significant differences (*p* < 0.05) among tissues for each specific gene, as determined by one-way ANOVA followed by Tukey’s HSD test. Bars sharing the same letter within a gene group are not significantly different from each other.

The expression levels of *PsopOBP* and *PsopOR* genes in the male and female antennal transcriptomes of *P. sophorae* were analyzed using FPKM values. Among these genes, seven PsopOBPs and the single identified *PsopOrco* were identified as significantly differentially expressed in male antennae (Table S4). Representative amplification and melting curves are presented in Figures S9 and S10, with complete quality metrics detailed in Tables S5 and S6. Notably, our expression analysis revealed sexually dimorphic patterns, with all eight target genes demonstrating significantly higher expression in male antennae versus female antennae. Specifically, *PsopOBP3*, *PsopOBP6*, and *PsopOrco* were exclusively expressed in the antennae, with *PsopOBP3* and *PsopOBP6* exhibiting more than a two-fold higher expression in male antennae compared to female antennae. Similarly, *PsopOrco* showed a more than four-fold increase in expression in male antennae relative to female antennae (Figure 4).

Additionally, several PsopOBPs were preferentially expressed in other tissues. For example, *PsopOBP8* (4.00 ± 0.26), *PsopOBP5* (4.71 ± 0.50), *PsopOBP4* (3.31 ± 0.15), *PsopOBP10* (10.42 ± 0.29), and *PsopOBP11* (5.53 ± 0.36) showed high expression levels in the head, legs, and thorax, respectively.

## 4. Discussion

In this study, we identified 11 candidate OBP genes and 11 candidate OR genes from the antennal transcriptome of *P. sophorae*. The diversity of OBPs and ORs across different insect species reflects significant variation, indicative of the wide range of adaptations in olfactory systems. While Diptera (40–90 OBPs) and Lepidoptera (50–60 OBPs) [16] exhibit a high number of OBPs, Hemiptera generally has a more limited repertoire, as seen in *P. sophorae* (11 OBPs), which is consistent with other scale insects, such as *D. corpulenta* (16 OBPs) [31] and *Ph. solenopsis* (15 OBPs) [30]. In contrast, the OR gene repertoire in coccid species, including *P. sophorae* (11), *D. corpulenta* (21), and *Ph. solenopsis* (1), is markedly smaller than that found in other Hemiptera, such as stink bugs (20–140), aphids (30–80), and planthoppers (30–150) (Table 3).

This study revealed that the number of OBP and OR gene family members in *P. sophorae* (11 each) is significantly lower than that in many other insects. We hypothesize that this streamlined olfactory gene repertoire may reflect long-term adaptation to its specialized ecological niche of feeding exclusively on licorice roots. The relatively limited diversity of odor cues in its subterranean habitat may have reduced the selective pressure for maintaining a large and diverse olfactory receptor arsenal. However, it is important to note that limitations of the current transcriptomic data (e.g., incomplete expression profiles for nymphal stages or specific tissues) imply the potential existence of undiscovered olfactory genes. Therefore, obtaining a complete genome sequence will be essential for definitively elucidating the full complement of olfactory genes in this species.

The number of classic OBPs identified in *P. sophorae* (6) is notably lower than that reported in other Hemipteran species (Table 3). Notably, ‘Plus-C’ OBPs, characterized by additional cysteine residues beyond the typical six, are absent in both *P. sophorae* and *Ph. solenopsis* [30], with only one ‘Plus-C’ OBP identified in *D. corpulenta* [31]. This pattern could be attributed to incomplete transcriptome sequencing in *P. sophorae*, a naturally low occurrence of ‘Plus-C’ OBPs in scale insects, or their genuine absence in this lineage. Among the eleven PsopOBPs identified, the six classic OBPs maintain the conserved cysteine pattern, while the remaining five exhibit variations in cysteine number and spacing. This diversity suggests potential functional divergence, possibly related to *P. sophorae*’s specialized ecological niche as an oligophagous root-feeding insect. The relatively simplified OBP repertoire in *P. sophorae* might reflect adaptation to its stable subterranean habitat with limited odorant diversity, reducing the need for a complex olfactory recognition system. Phylogenetic analysis of OBPs in *P. sophorae* and other hemipteran species revealed a clear correlation with taxonomic relationships. For example, OBPs from scale insects (*P. sophorae*, *Phenacoccus solenopsis*, and *Drosicha corpulenta*) predominantly clustered together in distinct clades separate from those of other hemipterans like aphids (*Acyrthosiphon pisum*) and mirid bugs (*Apolygus lucorum*). The phylogenetic analysis revealed distinct evolutionary patterns: the *PsopOBP7/8/9* cluster suggests recent gene duplications, while other PsopOBPs showed interspecific conservation. This pattern aligns with the ecological principle that some OBPs conserve general odor detection functions, while others diversify under species-specific selective pressures [16,68]. In *P. sophorae*, the divergent OBPs may represent adaptations to its unique host plant (*Glycyrrhiza uralensis*) and subterranean habitat, potentially enabling detection of root-specific volatiles or conspecific communication signals.The expression profiles of these genes provide further insights into their potential biological roles. OBPs have diverse functions across various insect tissues [50,65,69,70,71], with widespread expression in non-olfactory tissues being a common feature [72,73]. Beyond their canonical role in solubilizing and transporting odorants in antennal sensilla, OBPs are involved in chemosensation in mouthparts, egg-laying site selection by ovipositors, nutrient sensing in digestive organs, and even in visual signal modulation in compound eyes [16,74]. For example, certain OBPs expressed in legs contact host plant surfaces during walking, potentially mediating gustatory recognition of non-volatile compounds [75]. While numerous antennal OBPs have been demonstrated to bind volatile compounds in vitro [76,77,78], their in vivo functions extend beyond general odorant transport to include specialized roles such as: (1) selective transport of host-specific volatiles, and (2) precise pheromone detection and discrimination [79,80]. This functional specialization highlights how antennal OBPs may evolve distinct binding characteristics to support species-specific olfactory behaviors, rather than serving merely as passive solubilization carriers. Such non-olfactory functions may relate to processes such as nutrient transport or insect pheromone synthesis. In *P. sophorae*, *PsopOBP3* and *PsopOBP6* show antennae-biased expression, while *PsopOBP4*, *PsopOBP10*, and *PsopOBP11* are predominantly expressed in the thorax, *PsopOBP5* in the legs, and *PsopOBP8* in the head, indicating functional diversification of these OBPs across tissues [81,82,83].

Insects rely on two primary receptor types for detecting external odorants: the specialized odorant receptors (ORs) and the more conserved odorant receptor co-receptor (Orco) [84]. While ORs show substantial diversity and low sequence homology across species, each insect typically expresses a single Orco, which plays a similar role in olfactory recognition across species [85]. Notably, our structural prediction of *PsopOrco* suggests a potential deviation from the canonical seven-transmembrane (7TM) topology that has been widely reported for Orco proteins across numerous insect species, including *T. elegans*, *H. halys*, *Pl. stali*, and *C. Ciliata* (Figure S8). While the 7TM architecture appears to be highly conserved in most insect lineages, the predicted six-transmembrane structure in *PsopOrco* may represent a unique structural adaptation in scale insects. However, we cannot exclude alternative explanations such as limitations in transcriptome assembly or gene prediction accuracy. Future studies employing membrane topology assays or structural biology approaches will be essential to validate this observation and determine whether it represents a true evolutionary innovation in *P. sophorae*. Phylogenetic analysis of ORs revealed an evolutionary pattern analogous to that observed in OBPs: *PsopOrco* formed a conserved clade with Orco orthologs from other Hemipterans, while the remaining PsopORs exhibited significant diversification. Notably, *PsopOR4* and *PsopOR1* showed specific clustering with *DcorOR16* and *DcorOR17*, respectively (both with high bootstrap support). This conserved/diversified dichotomy suggests fundamental evolutionary principles governing chemosensory gene families in *P. sophorae*. However, we acknowledge that the current phylogenetic structure may be influenced by the limited taxonomic sampling of scale insect ORs, and expanded genomic data from closely related species could refine these cluster patterns. This suggests a complex evolutionary pattern where certain ORs maintain cross-species conservation while others develop species-specific divergence, reflecting differential evolutionary pressures [27]. RT-qPCR analysis confirmed that *PsopOrco* is highly expressed in the antennae, emphasizing its critical role in the olfactory system. Functional studies indicate that Orco does not function independently in odorant recognition; instead, it forms heteromeric complexes with traditional ORs, helping localize these receptors on the dendritic membranes of olfactory neurons and enhancing their sensitivity to odorants [86,87].

## 5. Conclusions

This study conducted the first systematic transcriptomic analysis of antennae from both male and female *P. sophorae* through high-throughput sequencing, identifying 11 odorant-binding proteins (OBPs) and 11 olfactory receptors (ORs). The research revealed eight chemosensory genes (*PsopOBP3/4/5/6/8/10/11*, *PsopOrco*) with male-specific overexpression patterns, suggesting their potential involvement in regulating sex-specific behaviors (e.g., courtship), though their specific chemosensory functions require further investigation given the current lack of chemical ecology data in *P. sophorae*. These findings not only provide molecular insights into the unique olfactory mechanisms of hemipteran insects but, more importantly, identify the unique domains of *PsopOrco* and male-specific olfactory pathways as potential molecular targets for developing targeted, environmentally friendly behavioral interference strategies against *P. sophorae* (e.g., designing novel disruptors or blockers), demonstrating clear potential for translating basic research into practical applications.

## Figures and Tables

**Table 1:** Information on the odorant-binding proteins (OBPs) identified in *Porphyrophora sophorae*.

Unigene ID	Name	ORF (aa)	Status	SP	BLAST Annotation	E-Value	Identity (%)	Score	Accesion
F-M_transcript_50750	*PsopOBP1*	317	complete	no	general odorant-binding protein 71 isoform X2 [*Cryptotermes secundus*]	1 × 10^−28^	30.09	122	XP_033606314.1
F-M_transcript_3768	*PsopOBP2*	193	complete	Yes	odorant-binding protein [*Phenacoccus solenopsis*]	6 × 10^−84^	68.98	257	ALS31064.1
F-M_transcript_33416	*PsopOBP3*	114	partial	Yes	odorant-binding protein 19 [*Dastarcus helophoroides*]	3 × 10^−22^	44.00	95	AIX97065.1
F-M_transcript_47028	*PsopOBP4*	181	partial	no	odorant binding protein 13 [*Drosicha corpulenta*]	2 × 10^−27^	40.00	111	ALV87607.1
F-M_transcript_7681	*PsopOBP5*	166	complete	no	general odorant-binding protein 56d-like isoform X5 [*Venturia canescens*]	6 × 10^−9^	30.65	63	XP_043267484.1
F-M_transcript_5182	*PsopOBP6*	156	complete	Yes	odorant-binding protein [*Phenacoccus solenopsis*]	1 × 10^−38^	42.67	139	ALS31056.1
F-M_transcript_3939	*PsopOBP7*	140	complete	no	odorant binding protein 3, [*Drosicha corpulenta*]	1 × 10^−9^	31.03	65	ALV87599.1
F-M_transcript_36157	*PsopOBP8*	116	complete	no	odorant binding protein 7 [*Drosicha corpulenta*]	2 × 10^−14^	40.00	76	ALV87603.1
F-M_transcript_45404	*PsopOBP9*	109	partial	no	odorant-binding protein 5 [*Phenacoccus solenopsis*]	9 × 10^−6^	52.17	54	ALS31055.1
F-M_transcript_21658	*PsopOBP10*	82	partial	no	odorant binding protein 9 [*Drosicha corpulenta*]	2 × 10^−22^	58.82	93	ALV87604.1
F-M_transcript_31593	*PsopOBP11*	65	partial	no	odorant-binding protein [*Phenacoccus solenopsis*]	2 × 10^−17^	59.68	82	ALS31059.1

**Table 2:** Information on the odorant receptors (ORs) identified in *Porphyrophora sophorae* (TMD: transmembrane domain).

Unigene ID	Name	ORF (aa)	Status	TMD	BLAST Annotation	E-Value	Identity (%)	Score	Accesion
F-M_transcript_31952	*PsopOrco*	428	complete	6	olfactory receptor protein 1 [*Phenacoccus solenopsis*]	0	96.14	920	ANW12106.1
F-M_transcript_35094	*PsopOR1*	493	complete	7	odorant receptor 17 [*Drosicha corpulenta*]	0	69.17	704	ALV87632.1
F-M_transcript_51349	*PsopOR2*	239	partial	2	olfactory receptor protein 2 [*Phenacoccus solenopsis*]	1× 10^−12^	31.51	77.8	ANW12107.1
F-M_transcript_37729	*PsopOR3*	108	partial	2	odorant receptor 8 [*Drosicha corpulenta*]	5 × 10^−9^	42.31	64.3	ALV87623.1
F-M_transcript_14069	*PsopOR4*	65	partial	0	odorant receptor 16 [*Drosicha corpulenta*]	2 × 10^−13^	55.38	75.1	ALV87631.1
F-M_transcript_47139	*PsopOR5*	64	partial	0	odorant receptor 4-like [*Aphis craccivora*]	1 × 10^−5^	36.67	52.4	KAF0765716.1
F-M_transcript_45891	*PsopOR6*	102	partial	0	odorant receptor 20 [*Drosicha corpulenta*]	2 × 10^−5^	39.34	51.6	ALV87635.1
F-M_transcript_31667	*PsopOR7*	83	partial	0	odorant receptor 5 [*Drosicha corpulenta*]	2 × 10^−13^	41.46	74.3	ALV87620.1
F-M_transcript_37558	*PsopOR8*	71	partial	0	odorant receptor 12 [*Drosicha corpulenta*]	5 × 10^−12^	45.45	66.2	ALV87627.1
F-M_transcript_44253	*PsopOR9*	160	partial	3	odorant receptor Or2-like [*Schistocerca cancellata*]	1 × 10^−14^	28.67	79.3	XP_049767968.1
F-M_transcript_47849	*PsopOR10*	387	partial	6	odorant receptor 59a [*Drosophila virilis*]	3 × 10^−6^	21.12	62.0	XP_002050827.1

**Table 3:** Quantitative statistics of odorant-binding proteins (OBPs) and odorant receptors (ORs) identified in Hemiptera species.

Species		OBPs				ORs	References
		Classic OBPs	Plus-C OBPs	Minus-C OBPs	Total		
Scale	6	0	0	11	11	11	This study
	10	1	0	16	16	21	Gao, 2016 [31]
	15	0	0	15	15	1	Zhao, 2016 [30]
Leafhopper	19	21	0	40	40	12	Bian et al., 2018 [45], Lun et al., 2023 [46]
	15	5	0	20	20	1	Chang et al., 2022 [15], NCBI
Stinkbug	12	4	0	16	16	24	Cui et al., 2017 [47], Cui, 2016 [48]
	26	12	0	38	38	110	Yuan et al., 2015 [49], An et al., 2016 [25]
	14	3	0	17	17	88	Sun et al., 2017 [50], Gu et al., 2011 [51], Xiao et al., 2017 [52]
	32	12	0	44	44	138	Sun et al., 2020 [53]
	14	5	0	19	19	121	Song et al., 2018 [54], NCBI
Aphid	13	2	0	15	15	79	Zhou et al., 2010 [55], Smadja et al., 2009 [56]
	7	2	0	9	9	33	Wang et al., 2019 [57], Liu et al., 2022 [27]
	9	0	0	9	9	45	Gu et al., 2013 [58] Cao et al., 2014 [59]
Planthopper	8	2	0	10	10	141	Zhou et al., 2014 [60], He et al., 2018 [61]
	-	-	-	9	9	37	NCBI, He et al., 2018 [62]
	8	3	1 Atypical OBPs	12	12	135	He & He, 2014 [63], He et al., 2015 [64]
Psylla	11	1	0	12	12	7	Xu et al., 2019 [26]
	7	2	0	9	9	46	Zhang et al., 2020 [65], Chen, 2016 [66]
Whitefly	7	0	1	8	8	-	Wang et al., 2017 [67]

## Data Availability

The data presented in this study are available in Supplementary Material. The raw sequencing data are currently not publicly available due to their relevance to ongoing research but can be made available upon reasonable request to the corresponding author.

## Supplementary Materials

The following supporting information can be downloaded at https://www.mdpi.com/article/10.3390/biology14101442/s1. Table S1: The primers information of genes in this study; Table S2: Summary of full-length transcriptome sequencing production; Table S3: Summary of functional annotation result; Table S4: Expression levels in the transcriptome of the male and female antennae of OBPs and ORs in *P. sophorae*; Table S5: Reference gene CV (%); Table S6: Standard deviation (SD) of Ct values; Figure S1: Consensus isoforms read length distribution. The abscissa refers to the consensus isoform sequence length distribution. The left ordinate refers to consensus isoform sequence length frequency distribution histogram. The right ordinate refers to consensus isoform sequence length cumulative frequency curve; Figure S2: Completeness assessment of the non-redundant FL transcriptome; Figure S3: Distribution of NR annotated species. All non-redundant transcripts of *P. sophorae* were used in tBLASTx to search the GenBank entries. The best hits with an E-value = 1.0 × 10^−5^ for each query were grouped according to species; Figure S4: Distribution of GO terms for all annotated transcripts in cellular component, biological process and molecular function; Figure S5: COG annotation of transcript sequences; Figure S6: eggNOG annotation of transcript sequences; Figure S7: KOG annotation of transcript sequences; Figure S8: Amino acid sequence alignment of *PsopOrco* with representative orthologs. Abbreviations (accession number in parentheses): Psol, *Phenacoccus solenopsis* (ANW12106.1); Dvit, *Daktulosphaira vitifoliae* (XP_050548961.1); Msac, *Melanaphis sacchari* (XP_025205994.1); Tele, *Tropidothorax elegans* (QQP19790.1); Ccil, *Corythucha ciliata* (AWW05228.1); Hhal, *Halyomorpha halys* (XP_014279420.1); Psta, *Plautia stali* (BCU40953.1); Figure S9: Amplification Plot of OBP4; Figure S10: Melt Curve of OBP4.
