# Supplementary material for: Identification and Characterization of Olfactory Genes in the Cochineal Scale Insect, Porphyrophora sophorae (Hemiptera: Margarodidae)
Source: Biology (Basel). 2025 Oct 18;14(10):1442. doi: 10.3390/biology14101442 (PMC12562052; doi:10.3390/biology14101442)
Supplement: Supplementary file 1 [file biology-14-01442-s001.zip › Supplementary Data File 1.pdf]

>PsopOBP1
MKKGCGIFRSRVFNLFILFVQLSNIQTLKCRTREQVEQQTDFYGLASDCMRTSMNLTKFS
SEDSTRGSRNSRHPQQGPMYSRRGGWNNNEQQREENGEFEGNSNRGRQRNGNNRRYKVHN
TGNNQCEPGQDDTRNPFENFRDEEKQGNQQRARIPNRYNQRDKRSTLILSKLNGKNSTRQ
NNIKSPLQDINSCVIHCIFREMNMLNSASQPDRSSMSKIMAGKIQNSELQEFIQDSIDEC
FDILETENDSANEEDKTCDFSKRLLLCLEEKAQRGCEDWNEDEDFKLFRGDEEPHDAYRS
NRNANNKMIGKQFGRG

>PsopOBP2
MKILSLILSLFVVCNLHVSINGEEAASTATPRCKAPTVAPQKLERIIGQCQDEIKSLLLQ
EALDVIGIGGQEFEVIKQQTARPKRELAASFSNEERRVAGCLLQCVYKKVKAVDDDGFPV
SEGLVRLYSEGVQDRNYFIATLTAVQQCVSIAEAVRQQQPNQKFDGGQTCDLAYEMFDCV
SDKIDKYCGVAA

>PsopOBP3
MFSRRNIAFSSFALILVTTVVIQEGACDELEEMMKALHETCVGQTGVSEDLIKQANAPGD
LPSDGKLKCYMKCLLSEVGVMDEDGEIDLDVLIDLLPEKVKADAQPVFDKCRD

>PsopOBP4
GGTDCFLANSRILGPVFYSVLLKFCYLITKFFIMKLLGVITLVAAFVCYAQAAESPELVE
KKSQVLNACKEELKPSEESLKIISTADIPSTEEQRCLLECIYKQVGMIKDGQLNKDGASG
IAKLRYGSEPEKLEKVEKIFDSCKGSIVADPAEKCSLGRKVRECVFKHPDGAQLRLFGNK

>PsopOBP5
MLWKVIILLTFFHIYCAVCLMQVADNYVLECNTTFPVKNYVIQRLKVEDLQNADRNLKCF
LQCALTKHGLFTAKGGVKIPDVPRDGVEPEQFKKMQQTLQNCSHSESQDGCDKVYDIVFC
YTKNIQDKMQITSEEETPSTDASLRDMERAVLETNFTSLEYSNLL

>PsopOBP6
MNTIFILVLSCSVLGLTYGQDVAHDVAKYVLDAEQKCIGNFPSITMEFLNNIKKQKKIPT
DPTQDFKCFLACLGTELSVMESSGNFNAEVLKQITKDITGGDVSHDEAHGMVSKCLEEIK
AEDECEMAFQYVLCKAKELEAFVFHKSFCPTCHDE

>PsopOBP7
MASFSHLAIMFALCFATISADTYEEAISHCKTQYKTDDAPLDQFLATGSFPAGKEDLKCF
FECLSQHLLIADAHGNLNQEAAKKVIEENQGKNPELGLFQEEVIKQCASPSSSSSACDKM
YEFVKCAVELKKAQAAPAK

>PsopOBP8
MEQAFFDCMVEYPTNQNIAAFFVNGAIPDESDHNAKCLFFCTPFKAGMVDEAFNINVQLA
KEWFFERHPGEKDGPHIDEALEECITKHKGEADHCEKAYVITKCLKKKNDEAKNS*

>PsopOBP9
EGYYFTKRYNLEVFRVYISRGTVSSFDMSRFTRSIVFFSTCFAFSNCLVPMEQAFFDCMV

EYPTNQNIAAFFVNGAIPDESDHNAKCLFFCTPFKAGMVKRTTVKKHT

>PsopOBP10
EGQQEIGAKAMEKCVKAKYVQWPDNEPGLKCNPMYVQFQHCLWKEYEMNCPEEERKDSKK
CTKLRKYLKQDGTSQNKNKDV

>PsopOBP11
AMTLAKQRFQENKDMLSKAETLFKTCKSEVEAAKSESERCVLGRLIRTCIVNNGKDVYIF
NKYE

>PsopOrco
MQKVRKTALVADLWPNIRLMQMSGLFFSAYHEDNSTMMRFIRKTYSWITTILMFTQYIFL
TIFAVTESYDADQRAAGAVTVLFFTHCLIKFTFFNMKSHSFYRTLNSWNNANSHPLFTES
NARHRAITLSRMRKLLYVIGTITIFSTVSWTGITFVGDSVREIPDPESENGTIVIDAPRL
MVPSYYPWNSMSGVGYIGALIYQFYWIFICLSIANLCDIMFCSLVIHACEQLKHLKEIMG
PLIELSAALDTQVNNTENLFRAASGGSKTALIEDGEYDNFAAGGNYMNYRSNVAGGAGPN
GLTKKQEILVRSAIKYWVERHKHVVRYVAMLTDMYGVALLLHMLITTFLLTILAYQATKS
SSVMEAAYSCQWYDGSEEAKTFVQIVCQQCQKALMVTGAKFFTISLDLFASVLGAVVTYF
MVLIQLN

>PsopOR1
MNYALVIFNWLFHSYEEFCELNLTYFKLFGLFQIVNPYNRVSERSQKIWLHAHGFITILT
GLHTMTQLVSLFVAQLSTSQIINNFTHIGNWSIAALKFANFAYNQKRLQWLFDILRVDFL
KVSKEQEYKQILYDNAYRCNWFILLYVFVCLMTTLVWNVYPMAELIYYSFLYVHIKNNHT
DYRPQSILDSWYPFETSEPPLYYWIYTYEFITYAYMLFAFCCYDSYLFSMFTMMTAQFYV
LTESFSNIGYDRPIRGYDPHLDNLFLFKSEKTLDIIREPLFKSINSECRVDNLALLKTEQ
LLDKEHHGRVIHLKGAEESEKAIEFHRNLVECVIDLQKANRYANELIEVYNDVIVFQVLV
SIVTICLMAFQSTIYFSKIGDDEFKGTFQISKCLLYFGCAFLLLYLSCLCSGDLNDARKG
INNAFYNAHWHEFQLPDMLKKDLLFSMTCGQKTHVVEITHIFPVDLHTFSEITEICFSYF
TFLLNYYKKKGL

>PsopOR2
EGLIRKHDIFTNISIILNVFTYWVCFSTPVITYIRRRELRQLFELIDTGFVKYNREDTVT
EENTEKKLRAFLSLILKLTAGAYLFLLLIPLVLAYSSSDQRTKTGFDLLYYAWVLFDINR
YYLLNYFTQIVATTSLYFAYNTKCLAVLLYVSELKFQCDRYHSALRHICDRCYKKCVLKP
TDERTKGMSNSAIVKCDEGQFIRNLKYSQIHYNQIRRSVTFISRSPIITFLSAQNRSY

>PsopOR3
MIFYSLLKSELNVAFLNYSMIQIFGFLNCCYSGEVFLSLNAMLCEALYGTNWYEYSVENR
RFLLTFISQLATPLHIKGWKISILSLNMFVRILRTSYSYFNVLRTVY

>PsopOR4
GVYNIPWYSASIRVRKGVLTLLMQTQYEYQMKAFGMFPVNRGLFAKVLKLNFSLLNLTLL
VYSR

>PsopOR5
MLYSVEWWLLPSRHRKSMLIALTRSNQPVQLKFGVSFIAGFPLLVGILKNAYTYANFFKT
LNN

>PsopOR6
MKEYTSAFTLQFQMALCCSSLMFALQTYRLVQNNTVVNMLYSTEWWVQPVRCPKLILIIM
TRSTDAVQLKFGVHFIAGYSLLVGILKNAYSYSNFLNKFNV

>PsopOR7
MYCYAGETFRDVNDVFREAVYNMEWYETSIEFRKLLLLLLTRTQKELNLQFSMKYVASYA
TFASVLKASYTYASFLLNVINK

>PsopOR8
MSDEIRFSIYCCDWIGCSPHLLKYMIIMMSSVERPLKLKIFMVSEASLSTFTNMVRLSFS
YFNVFRNLLK

>PsopOR9
EGTAIDDLTTCVKCHHKLLRLWKSVQRFLYAYLLVELCAVVVMMSLTYFKLSLVTDASDL
VMDYCFVGCVTVWVFLDCCSGSLVEEMNERIRFSLYSCEWINCSPRSRKFIVIMMSSVER
PLKLKVPMVSEASLPIFCSMMKLSFSYYNVLRRVQHTMR

>PsopOR10
GETYRCSSEGSIMSYKFSEMLTETGKSSPRKGGAKRRLLTWLNRICLDIGVQLNPNDDFS
RTKKLYANTKLLCAALVGIGSLILLCSSKDLTSTLNNVRGVLVCILLSVFYATYIARIQD
CRMIFHMLTYECAFEFEQREKNIFYSVSEKMGKIIAAYVISIPMFGVVIMQFPMWLALWN
NFPREWEKHLFIPIWVPENDSILTTYMVFCISVVGNLTILEIAITKTMLSLASFLIAAGE
FHALHCHVDEINADNLWRNDVGEQISVDRLTCCVKWHSNLLKLWKHVEGFCGVVCMIEFI
YAVLAISQTYLKLSLATDITDFTIDIAFAIGVSIWIFPECFAGSFVEELNDEVRFSVYCC
DWTSFSPKLRKTHNHYDELHATSVEA
