# Supplementary material for: Identification and Characterization of Olfactory Genes in the Cochineal Scale Insect, Porphyrophora sophorae (Hemiptera: Margarodidae)
Source: Biology (Basel). 2025 Oct 18;14(10):1442. doi: 10.3390/biology14101442 (PMC12562052; doi:10.3390/biology14101442)
Supplement: Supplementary file 1 [file biology-14-01442-s001.zip › Table Suppl.pdf]

Table S1. The primers information of genes in this study

Gene	Forward primer	Reverse primer
PsopOBP3	GGACAAACTGGAGTCTCTGAAG	CCTCGTCCATCACTCCTACT
PsopOBP4	CGAGCCTGAGAAACTAGAGAAG	CACATTCACGAACTTTCCTTCC
PsopOBP5	TAGCGAAAGTCAAGATGGATGTG	GCGTTTCTTCCTCACTGGTAAT
PsopOBP6	GCGGAAGTACTGAAGCAGATTA	GTACATACTGGAACGCCATCTC
PsopOBP8	GAGAAAGACGGTCCGCATATAG	GGAGTTCTTCGCTTCATCGT
PsopOBP10	GATGGAAAAGTGCGTGAAAG	CACAAGCAATGCTGAAACTG
PsopOBP11	GCAATGACACTAGCTAAGCAAC	GATTCAGACTTTGCCGCTTC
PsopOrco	CGTACGATGCTGACCAAAGA	CAGGAGTTCAGGGTTCGATAAA
A-tubulin	CAAGCAAGAATACGACGAATCCG	CAGTACAACCACGGTCGCATTAA

Table S2. Summary of full-length transcriptome sequencing production.

Type	Count
Circular consensus (CCS) reads	412,849
Full length reads non-chimeric (FLNC)	372,326
FL consensus isoforms	51,769
High-quality FL transcripts	51,764
Non-redundancy high-quality FL transcripts	33,579
Alternative splicing	98
Simple sequence repeats (SSR)	3,745
coding sequences (CDS)	7,903
lncRNA	17,092
Annotation	16,568

Table S3. Summary of functional annotation result.

Annotated databases	Isoform Number
COG	5,114
GO	7,937
KEGG	9,454
KOG	11,720
Pfam	11,162
Swiss-Prot	9,504
eggNOG	15,106
nr	15,600
All	16,568

Table S4. Expression levels in the transcriptome of the male and female antennae of OBPs iand ORs in *P. sophorae*.

**Unigene ID**	**Name**	M1_FPKM	M2_FPKM	M3_FPKM	F1_FPKM	F2_FPKM	F3_FPKM	Regulated	p
F-M_transcript_50750	PsopOBP1	153.58	149.65	156.84	391.71	381.84	393.78	up	<0.05
F-M_transcript_3768	PsopOBP2	5.48	5.55	4.16	47.15	39.38	42.79	up	<0.05
F-M_transcript_33416	PsopOBP3	63204.01	64581.9	64382.83	6855.14	6380.12	7201.59	down	<0.05
F-M_transcript_47028	PsopOBP4	1934.03	2049.63	1967.41	621.53	601.4	619.54	down	<0.05
F-M_transcript_7681	PsopOBP5	893.18	959.78	890.33	235.82	218.1	226.26	down	<0.05
F-M_transcript_5182	PsopOBP6	92115.03	96005.62	94503.57	530.71	445.48	578.3	down	<0.05
F-M_transcript_3939	PsopOBP7	27.67	15.26	20.87	258.46	205.42	227.32	up	<0.05
F-M_transcript_36157	PsopOBP8	17.21	14.48	15.85	11.55	9.09	8.18	down	<0.05
F-M_transcript_45404	PsopOBP9	1.66	1.92	5.36	21.89	9.45	9.25	up	>0.05
F-M_transcript_21658	PsopOBP10	6.21	7.36	7.93	1.79	1.85	1.79	down	<0.05
F-M_transcript_31593	PsopOBP11	92.1	101.42	104.68	3.72	3.16	2.39	down	<0.05
F-M_transcript_31952	PsopOrco	308.85	302.23	322.41	65.31	62.08	61.25	down	<0.05
F-M_transcript_35094	PsopOR1	6.13	5.99	6.15	4.66	6.65	5.47	down	>0.05

F-M_transcript_51349	PsopOR2	0.79	0.14	0	1.27	1.19	1.24	up	>0.05
F-M_transcript_37729	PsopOR3	0	0	0	0	0.32	0.16	up	>0.05
F-M_transcript_14069	PsopOR4	3.09	12.13	11.88	0	0	3.42	down	>0.05
F-M_transcript_47139	PsopOR5	0	0	0	0	0.31	0.33	up	>0.05
F-M_transcript_45891	PsopOR6	0.36	0.62	0	0	0.67	1.05	up	>0.05
F-M_transcript_31667	PsopOR7	1.02	0.43	0.78	0.11	1.4	0.89	up	>0.05
F-M_transcript_37558	PsopOR8	0	0	0.5	0	2.95	1.1	up	>0.05
F-M_transcript_44253	PsopOR9	1.59	1.09	0.78	2.16	1.4	1.33	up	>0.05
F-M_transcript_47849	PsopOR10	2.11	2.03	2.32	2.49	3.34	3.24	up	<0.05

Table S5. Reference gene CV (%)

Sample Group		Mean CT Value	SD	CV (%)	Stability Conclusion
Antenna	male	23. 46	0. 20	0. 74	＜5%
	female	21. 21	0. 50	2. 27	＜5%
Head	male	23. 12	0. 10	0. 22	＜5%
	female	23. 19	0. 10	0. 35	＜5%
Thorax	male	23. 19	0. 05	0. 21	＜5%
	female	23. 12	0. 10	0. 42	＜5%
Abdomen	male	22. 98	0. 10	0. 39	＜5%
	female	23. 13	0. 20	0. 80	＜5%
Leg	male	23. 23	0. 10	0. 23	＜5%
	female	23. 12	0. 10	0. 38	＜5%

Table S6. Standard deviation (SD) of Ct values

Sample Group		SD				
		Antenna	Head	Thorax	Abdomen	Leg
PsopOBP3	male	0. 37	0. 15	0. 17	0. 03	0. 11
	female	0. 13	0. 42	0. 26	0. 38	0. 17
PsopOBP4	male	0. 24	0. 07	0. 09	0. 06	0. 13
	female	0. 10	0. 26	0. 09	0. 26	0. 07
PsopOBP5	male	0. 56	0. 09	0. 18	0. 12	0. 19
	female	0. 13	0. 28	0. 10	0. 38	0. 10
PsopOBP6	male	1. 01	0. 08	0. 05	0. 12	0. 12
	female	0. 09	0. 16	0. 17	0. 35	0. 21
PsopOBP8	male	0. 43	0. 14	0. 26	0. 18	0. 27
	female	0. 29	0. 21	0. 04	0. 16	0. 09
PsopOBP10	male	0. 39	0. 25	0. 08	0. 18	0. 06
	female	0. 06	0. 15	0. 09	0. 43	0. 13
PsopOBP11	male	0. 60	0. 25	0. 17	0. 03	0. 11
	female	0. 22	0. 32	0. 26	0. 38	0. 10
PsopOrco	male	0. 42	0. 05	0. 04	0. 17	0. 23
	female	0. 15	0. 25	0. 13	0. 16	0. 08
