# Supplementary figures and images for: Identification and Characterization of Olfactory Genes in the Cochineal Scale Insect, Porphyrophora sophorae (Hemiptera: Margarodidae)
Source: Biology (Basel). 2025 Oct 18;14(10):1442. doi: 10.3390/biology14101442 (PMC12562052; doi:10.3390/biology14101442)

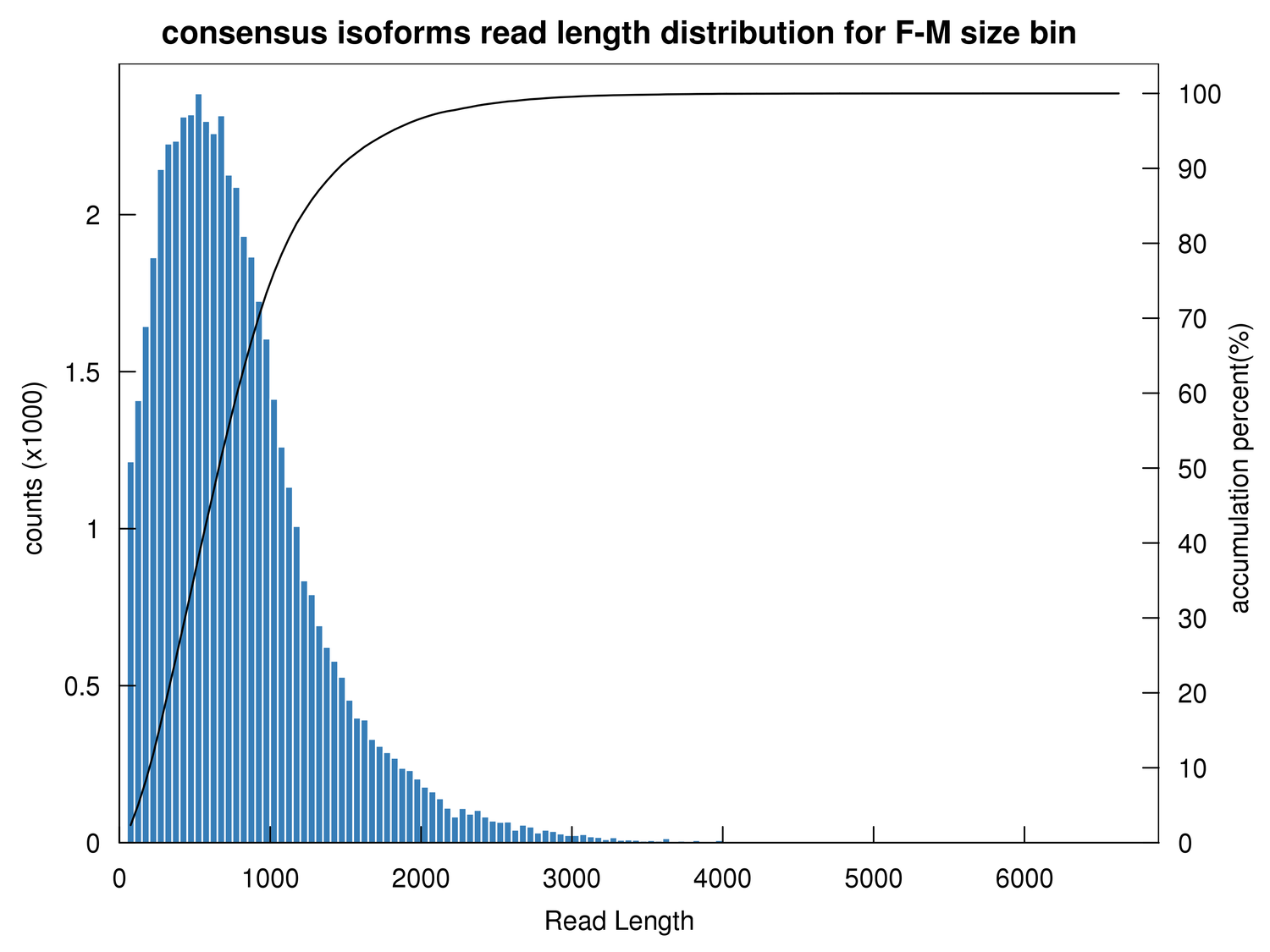

Supplement: Supplementary file 1 [file biology-14-01442-s001.zip › Figure Suppl.1..png]

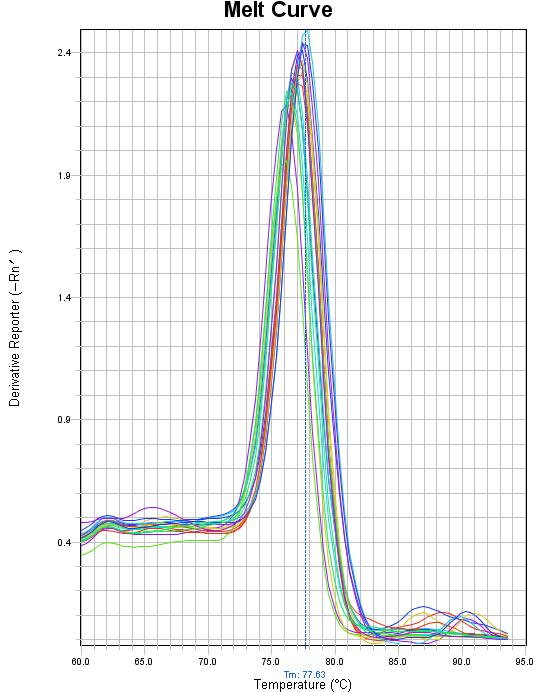

Supplement: Supplementary file 1 [file biology-14-01442-s001.zip › Figure Suppl.10 Melt Curve OBP4.jpg]

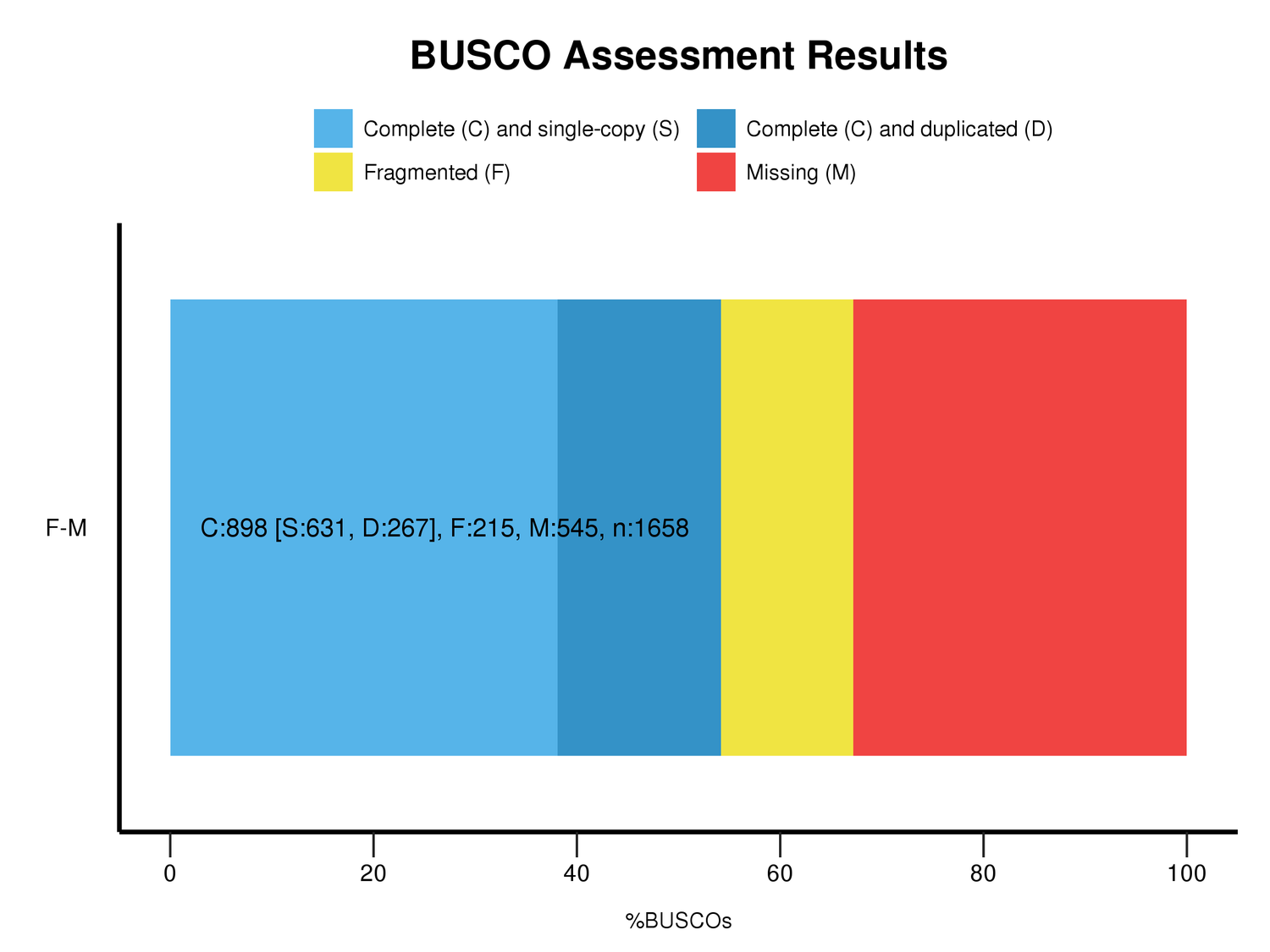

Supplement: Supplementary file 1 [file biology-14-01442-s001.zip › Figure Suppl.2. .png]

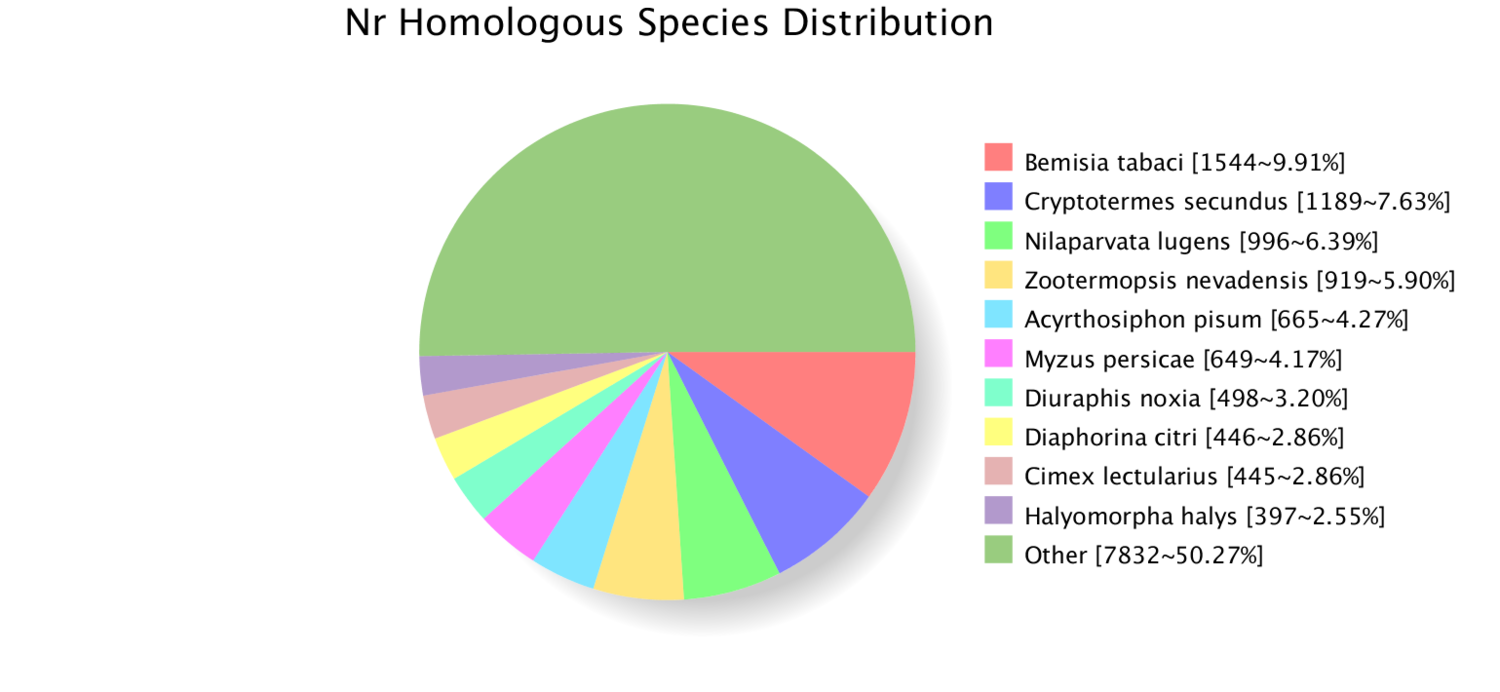

Supplement: Supplementary file 1 [file biology-14-01442-s001.zip › Figure Suppl.3. .png]

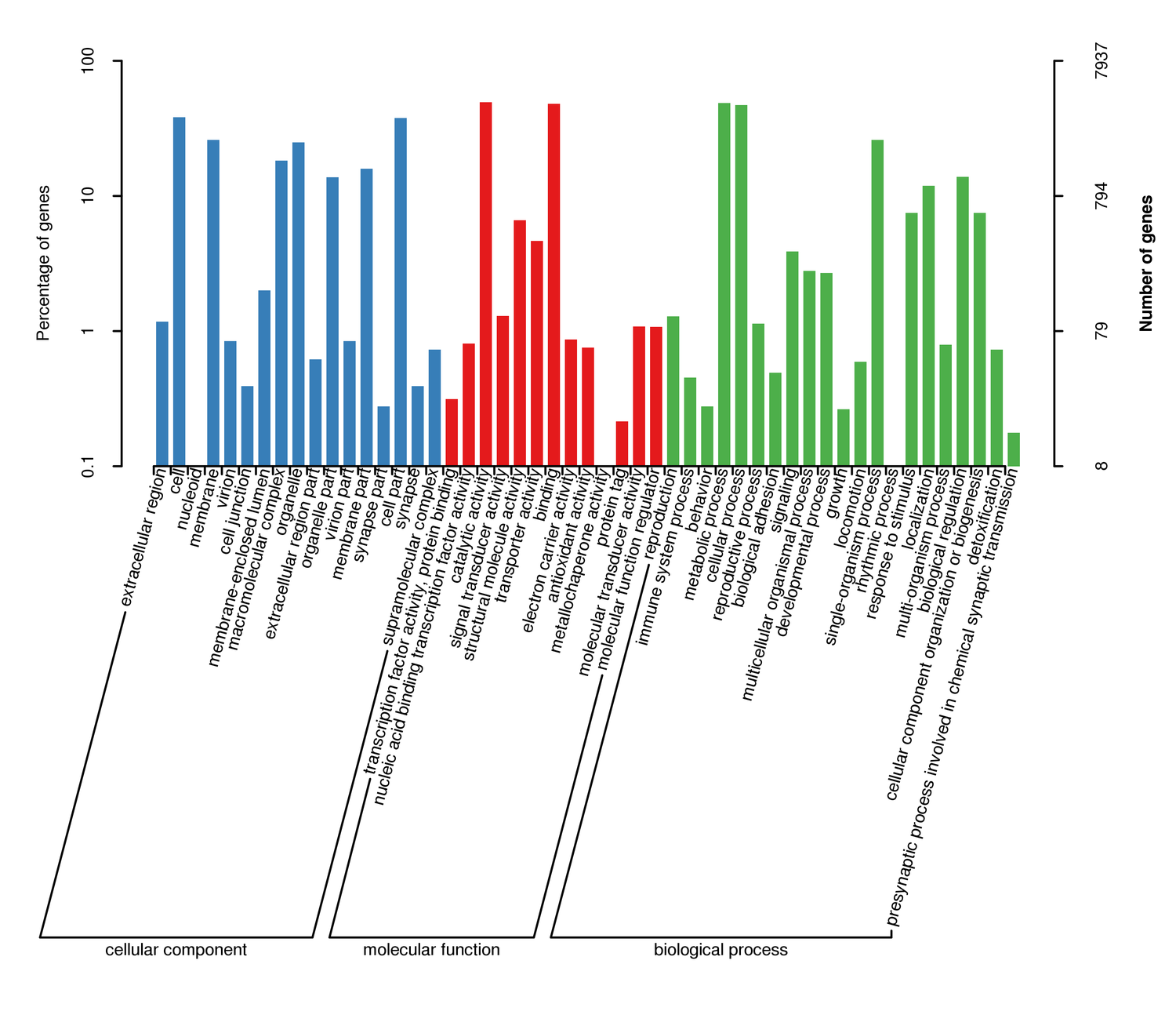

Supplement: Supplementary file 1 [file biology-14-01442-s001.zip › Figure Suppl.4..png]

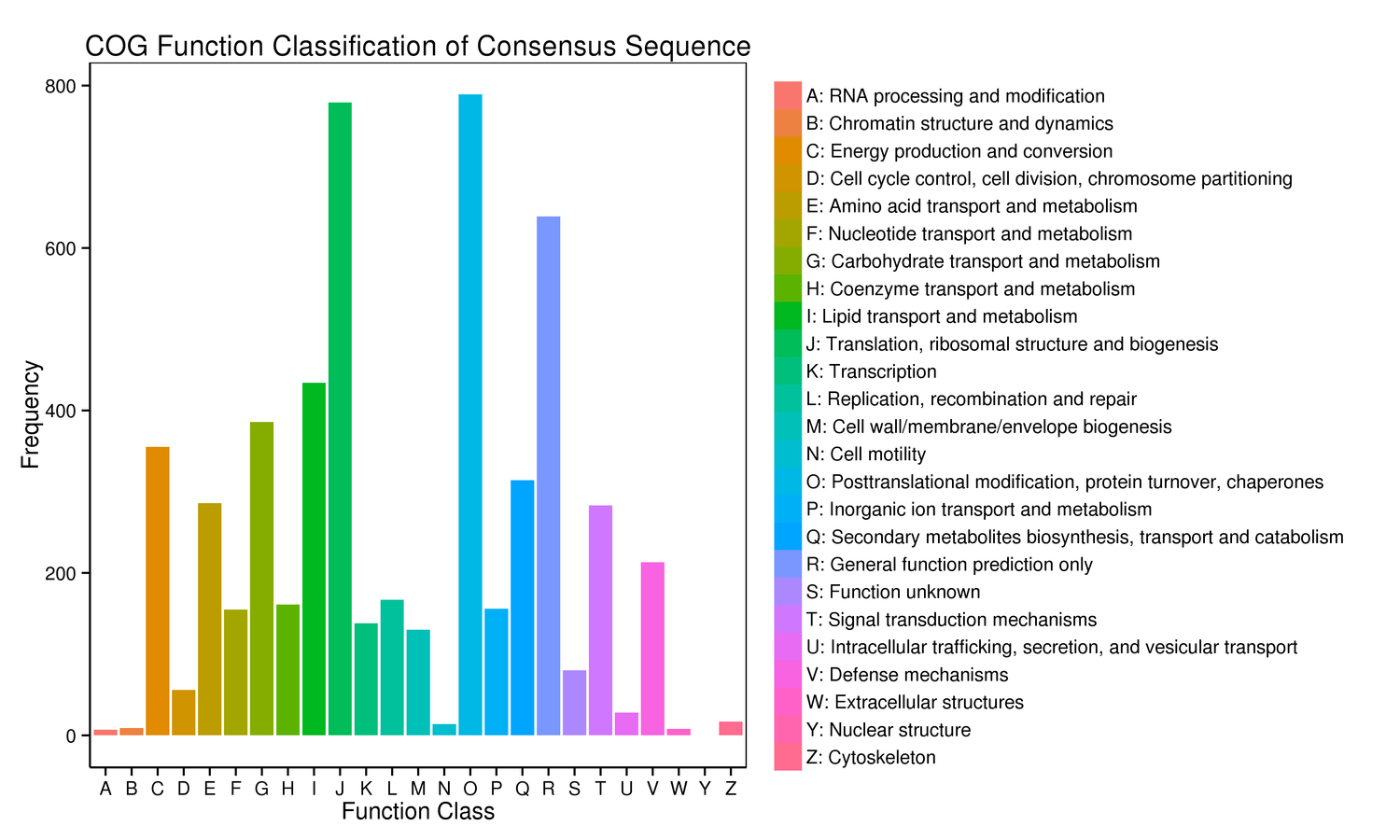

Supplement: Supplementary file 1 [file biology-14-01442-s001.zip › Figure Suppl.5..png]

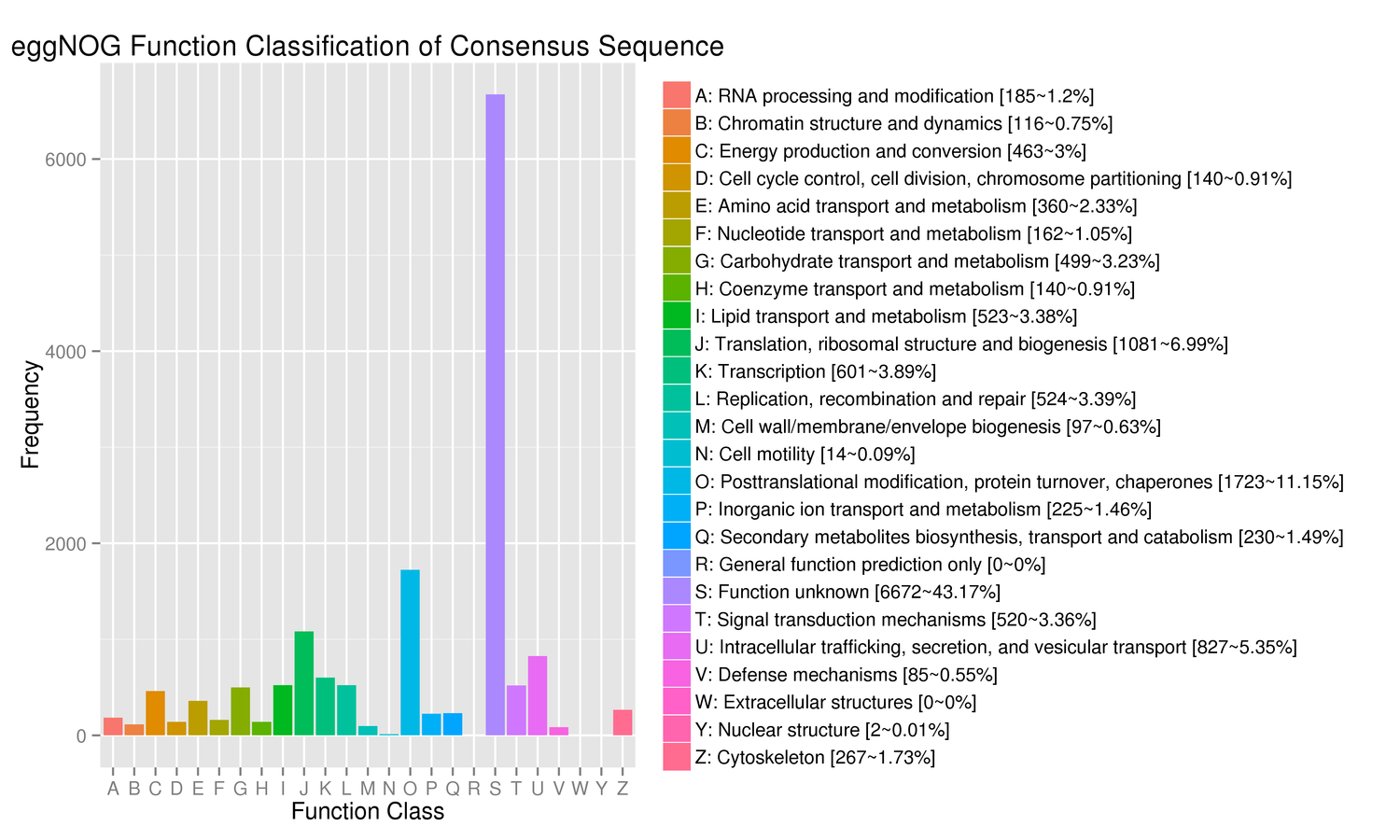

Supplement: Supplementary file 1 [file biology-14-01442-s001.zip › Figure Suppl.6..png]

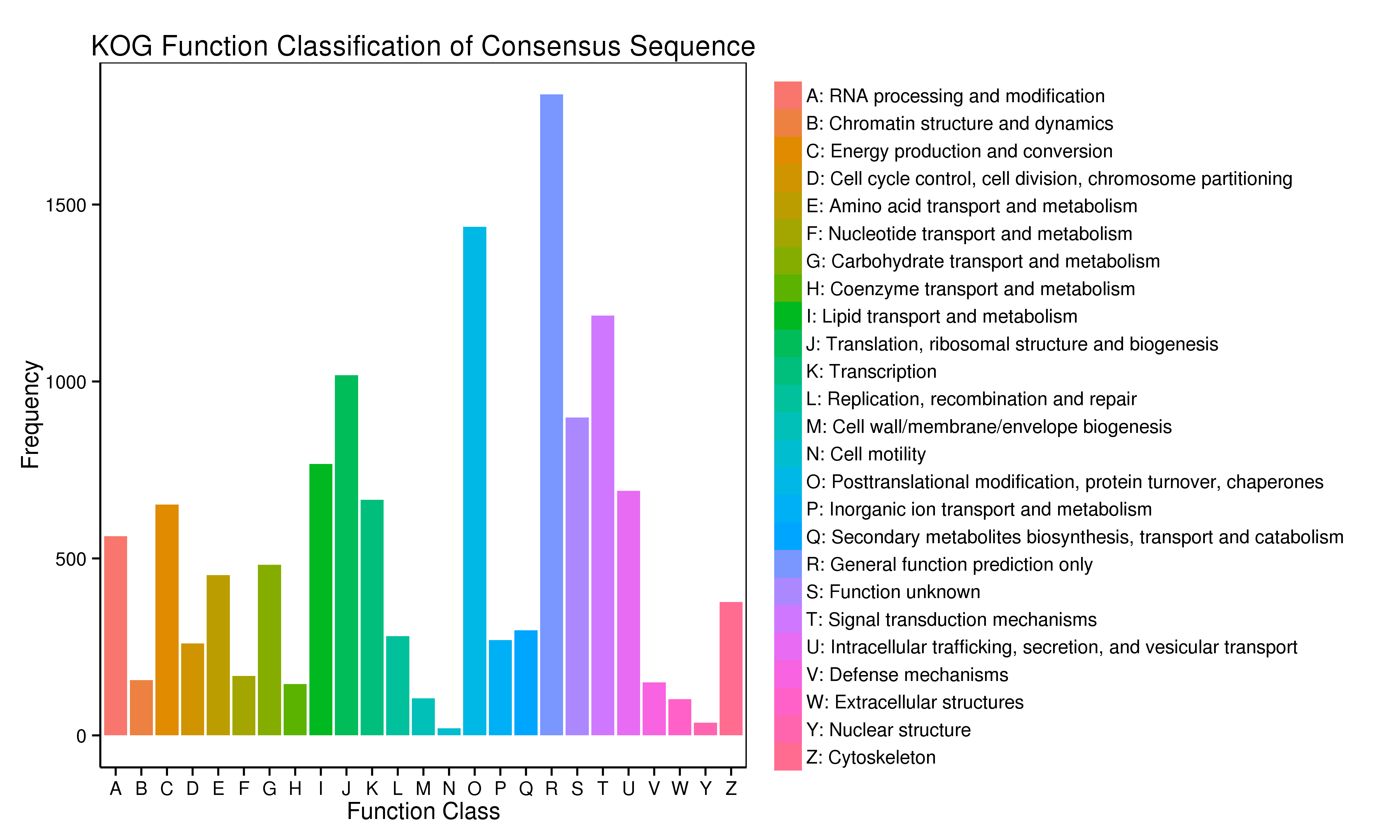

Supplement: Supplementary file 1 [file biology-14-01442-s001.zip › Figure Suppl.7..png]

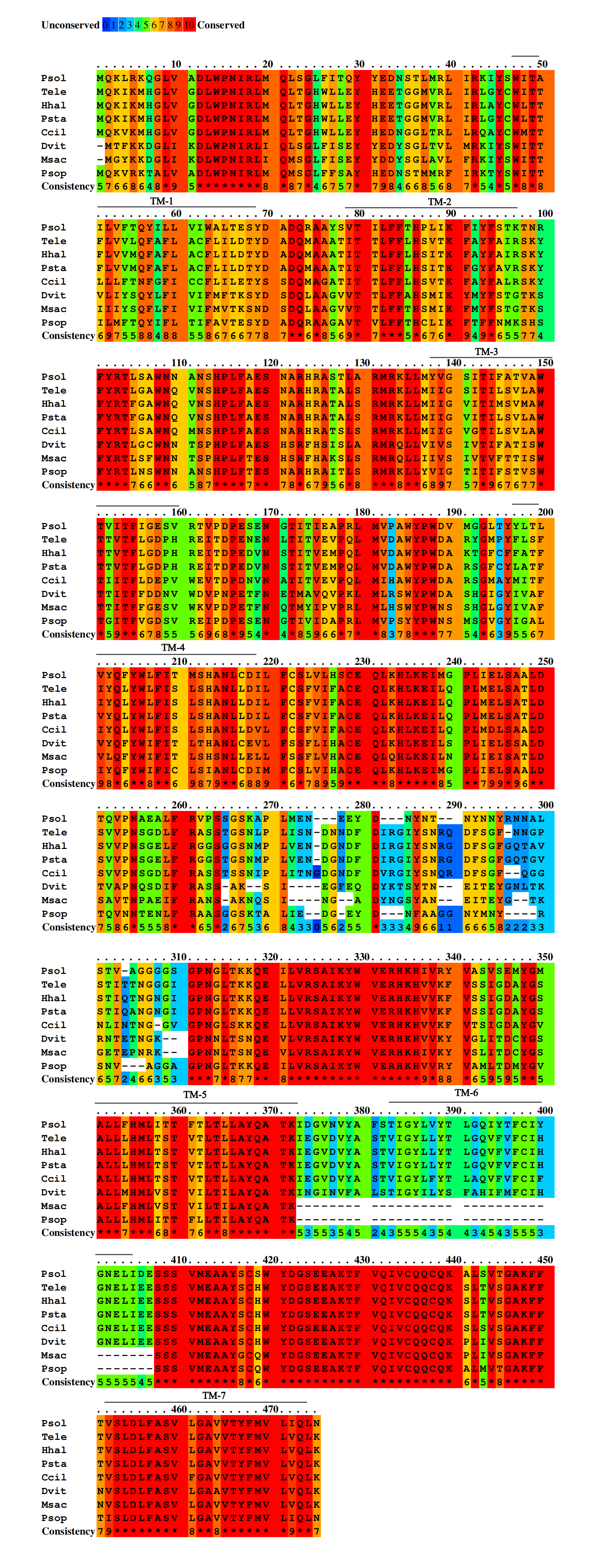

Supplement: Supplementary file 1 [file biology-14-01442-s001.zip › Figure Suppl.8.png]

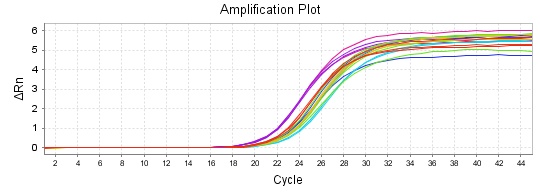

Supplement: Supplementary file 1 [file biology-14-01442-s001.zip › Figure Suppl.9 Amplification Plot OBP4.jpg]
